# Genome Sequence of the *Mycobacterium smegmatis* Bacteriophage Eugenia

**DOI:** 10.17912/micropub.biology.001401

**Published:** 2024-12-14

**Authors:** Vipaporn Phuntumart, Lucia Boulos, Bella Nunnally, Isabella Lima, John Motter, Olivia Sidoti, Sam Rutherford, Hsin-Ho Wei, Raymond Larsen, Jill H Zeilstra-Ryalls

**Affiliations:** 1 Biological Sciences, Bowling Green State University, Bowling Green, Ohio, United States

## Abstract

We report the discovery and genome sequence of mycobacteriophage Eugenia, isolated from soil samples collected in Akron, OH. Eugenia is a double-stranded DNA virus with a genome size of 69,139 bp, featuring 104 predicted protein-encoding genes, with 32 of these genes assigned putative functions.

**Figure 1. Transmission electron micrograph of Eugenia taken using Zeiss EM10:**
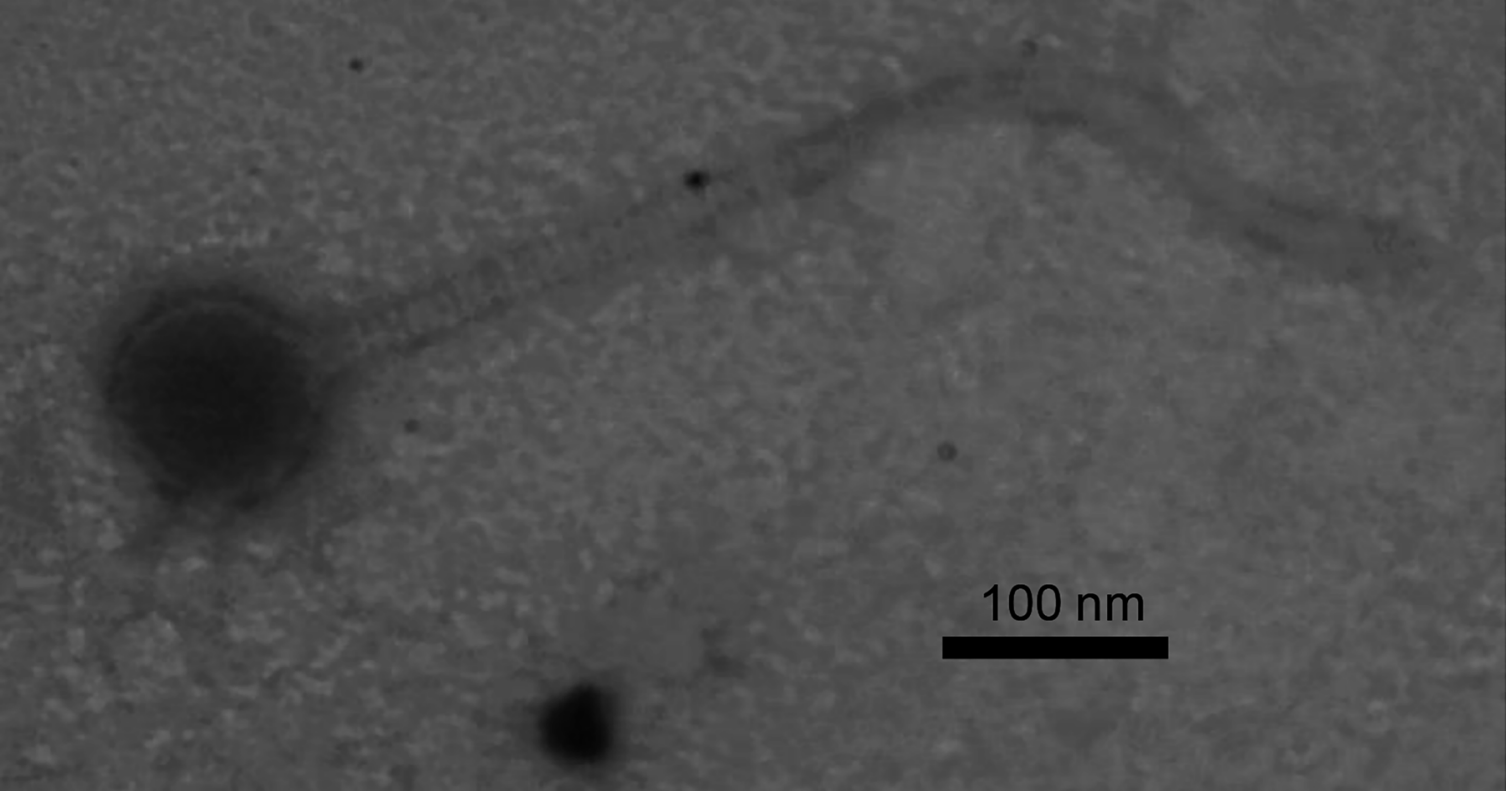
The phage was negatively stained with 2% uranyl acetate. Its capsid diameter is 75 nm and the tail length is 530 nm.

## Description

Bacteriophages have fueled fundamental discoveries in biology, the development of biotechnological tools, and therapeutics. The latter is highlighted by the recent treatment of a patient infected with multidrug-resistant *Mycobacterium abscessus* using a cocktail of phages that were isolated from the nonpathogenic bacterium *Mycobacterium smegmatis* mc *^2^* 155 (Dedrick et al., 2019; Mohan et al., 2015) . Here, we present the characterization and sequence of a new mycobacteriophage, Eugenia. This phage was isolated from soil in a flower bed in Akron, Ohio (41.1871 N, 81.6822 W), using an enrichment procedure ***(*** Zorawik et al., 2024). Briefly, the soil sample was washed in nutrient broth (7H9), the wash was filtered using a 0.22 *µm* pore size filter, and the filtrate was inoculated with *M. smegmatis* mc *^2^* 155. Following incubation in 7H9 media for 48 hr at 37˚C, the bacteria were removed by filtration, and the filtrate was plated in top agar with *M. smegmatis.* After two days of incubation at 37˚C, Eugenia phage were isolated from a clear plaque with a diameter of approximately 1 mm. Eugenia was then plaque-purified through five rounds of plating. Negative stain (2% uranyl acetate) transmission electron microscopy (Zeiss EM10) revealed Eugenia to have a siphovirus morphology with a capsid size of 75.2 ± 9 nm and tail length of 445 ± 57 nm.

Genomic DNA was extracted from a high titer lysate using the Promega Wizard DNA cleanup kit, and a library was generated using the Illumina Truseq Nano DNA prep kit. Sequencing was performed using a MiSeq System and v3 600 Cycle Reagent kit (2 x 300 paired end reads), yielding ~ 2,280,377 bp of 300-base single-end reads. The raw reads were assembled using Newbler v2.9 and default parameters achieving a coverage of 4,682 (Margulies**** et al., 2005 *).* Methods described by Russell (2018) were used to ensure the completeness and accuracy of the sequence.

The Eugenia genome was annotated using bioinformatic tools and databases that included DNAMaster v5.23.6 (Pope and Jacobs-Sera, 2018) embedded with Glimmer v3.02b (Delcher et al., 1999) and Genemark v2.5 (Besemer and Borodovsky, 2005) , PECAAN v1.0 (Rinehart et al., 2016; https://discover.kbrinsgd.org/), PhagesDB BLAST: Actinobacteriophage database (Russell and Hatfull, 2017) , NCBI BLAST: non-redundant database (Altschul et al., 1990) , Phamerator database v505 (Cresawn et al., 2011) , Starterator database v505 (Pacey, 2016; http://phages.wustl.edu/starterator/), HHPRED v3.2.0: PDB mmCIF70, Pfam-A (Söding et al., 2005) , NCBI Conserved Domain databases (Wang et al., 2022) , Aragorn v1.1 and v1.2.38 (Laslett and Canback, 2004) , tRNAscanSE v2.0 16. (Lowe and Eddy, 1997) , TMHMM v2.0 (Krogh et al., 2001) , and SOSUI v1.11 (Hirokawa et al., 1998) . Eugenia was assigned to cluster B, subcluster B1 based on gene content similarity of at least 35% to phages in the Actinobacteriophage Database (Russell and Hatfull, 2017) . Its genome is 69,139 bp, making it among the largest genomes of B1 subcluster phages, to date. It has 3′ single-stranded overhang of 9 bases (CGCGGGGGA) and a G+C content of 66.5%, which is similar to that of the host *M. smegmatis* mc² 155 (67.4%) bacterium (Mohan et al., 2015) . A total of 104 putative protein-encoding genes were identified, and of these 32 were assigned putative functions, without any tRNA. The DNA sequence of Eugenia is available at GenBank PP978882.1 and the raw sequence data have been deposited in the Sequence Read Archive (SRA) under No. SRX25258414 .

Phages of the B1 subcluster have therapeutic potential because they display inhibitory action toward biofilm formation and form plaques on strain 4XR1, an isoniazid-resistant derivative of *M. smegmatis* mc ^2^ 155 (Mohan et al., 2015) . While Eugenia forms clear plaques and is predicted to be lytic based on the lack of identifiable immunity repressor or integrase functions, phage-release experiments suggest that some cluster B1 phages may be temperate (Das et al., 2024) . A RepA-like protein in B1 phages that is also present in Eugenia (gp58) has been predicted to be involved in lysogeny (Das et al., 2024) ***.*** However, this protein bears no similarity to RepA-like proteins of mycobacteriophages whose function has been experimentally linked to lysogeny (Wetzel et al., 2020) . Nevertheless, it raises important questions about the mechanisms of action and potential applications of Eugenia in therapeutic contexts, warranting further investigation into its biology.
